# The Weight of Waiting

**DOI:** 10.3201/eid3204.251540

**Published:** 2026-04

**Authors:** Moussa Moise Diagne

**Affiliations:** Arboviruses and Hemorrhagic Fever Viruses Unit, Institut Pasteur de Dakar, Dakar, Senegal

**Keywords:** viruses, Ebola virus disease, Crimean-Congo hemorrhagic fever, outbreak response, laboratory diagnostics, epidemic ethics

## Abstract

In Ebola outbreaks, families wait to bury their dead until a PCR whispers yes or no. Amid outbreaks of hemorrhagic fever in Senegal, laboratories raced clocks they could not command as loved ones stood by. This essay explores the ethics and emotion of that fragile interval between sample and answer.

## Between Sample and Answer

Outbreak science is often told as a story of discovery: the virus identified, genomes sequenced, clusters mapped. In the field, however, science is not measured in breakthroughs. Science is measured in waiting. Waiting for a test result. Waiting for permission. Waiting for certainty before grief can proceed, before care can begin.

In the laboratory, time feels technical, predictable. Thirty cycles of PCR. Forty minutes of centrifugation. Six hours on a sequencer. Time in the lab is a sequence of protocols. Some of that waiting is integral, not out of indifference but out of care. In outbreaks like Ebola, guidance from international agencies and national authorities determines what must happen before an answer can travel (*1*): how a sample is handled and moved, how results are checked and released, and when families can lay their dead to rest. Such protections save lives, even as they stretch the human space between a sample taken and certainty delivered. Outside the lab—in a hospital ward, in a village courtyard, at a laboratory gate—time fractures. Moments are felt in the rising panic of families who want to bury their dead, in the restrained hands of clinicians at a patient’s bedside, in the quiet tension of how much longer waiting is possible.

This contradiction has followed my work as a virologist. My role has been to extract the truth from blood and tissue. In outbreak zones, I learned that the truth is not delivered in those controlled, sterile places in which it is revealed. Truth arrives against the backdrop of grief, fear, and trust stretched thin. Most often, it arrives too late for those seeking it.

### Scene 1—At the Laboratory Door (Beni, North Kivu, Democratic Republic of Congo, 2019)

The outbreak was already months old when I arrived in Beni, after an early stint in Mangina, where the first sparks had caught (*2*). In Mangina, confusion. In Beni, something heavier, grief hardened into suspicion.

One afternoon, a man died. His family was told to wait for the PCR result before burial. The epidemiologists and socio-anthropologists did the explaining. The family listened, at first. Then the hours stretched, elastic and cruel.

The delay was not owing to neglect. Samples poured in daily, from the living and the dead, and each tube demanded safe handling, logging, extraction, amplification. Couriers threaded insecure roads to bring samples in; machines ran almost constantly. Even so, the reality of field diagnostics often translates to abrupt nighttime pauses: patients arriving at night were typically sampled the next morning once the lab opened, and results were targeted within a day or two under difficult conditions (field laboratories tracked <48-hour turnaround for suspect samples during this outbreak) (*3*).

The family’s patience broke. They came directly to the laboratory, voices raised, fists pounding on doors. For them, waiting had crossed into injustice. For us inside, the moment was suffocating, caught between stress and comprehension. We could not deny their grief, but we could not conjure truth faster than the cycles would allow.

I remember standing by the glass, seeing their anger, hearing their shouts, while technicians in suits kept their heads down, processing samples one by one. Between us lay racks of tubes, each a story held at 95°C, denatured and re-annealed, amplified toward a verdict. In that corridor, I realized that waiting was a shared burden, but not an equal one. The family waited to bury. We waited to be sure. Everyone paid the price of that interval.

As the outbreak unfolded across North Kivu and Ituri, the world’s second-largest Ebola outbreak (*4*), field labs multiplied and testing volume surged. The World Health Organization’s situational reports from 2019 described 10–11 operational field labs and thousands of samples processed weekly despite conflict and access constraints (*2*,*5*). The technology promised speed; the terrain and insecurity rationed it. Families lingered overnight outside gates. Inside, we tallied cycles and prayed the power held. In the ledger of outbreak work, those hours accumulate like interest, emotional debt that someone, somewhere will have to pay.

The result eventually came. It always does. In Beni, though, as in so many places, the result arrived threaded with the memory of the wait, and with a thinner strand of trust.

### Scene 2—Curves on a Screen, Silence in a Ward (Dakar and Podor, Senegal, 2022)

Years later, I lived another form of waiting, not at the bedside, but at the Institut Pasteur de Dakar bench. Calls came from Podor, in the Saint-Louis region. Fever, bleeding, collapse: suspected viral hemorrhagic fever. Tubes began to land on our benches, tagged with urgency and carefully packed miles away, where families and clinicians were bracing.

We moved as fast as protocols permitted. Extraction. Master mix. Plates set, sealed, and run. Confirmation assays. Each step deliberate, each minute felt. We knew the ward rhythm by proxy: the pacing in a corridor, the weight of a clinician’s hesitation, the family watching the gate. In outbreaks, a lab result is never just data; a hinge turns on that result, and action swings.

That month, Podor learned Crimean-Congo hemorrhagic fever’s terms. Three human cases were confirmed; 2 patients died. We documented virus circulation in local ticks and livestock. It was the first severe cluster of its kind there, and it cut fast (*6*). We delivered results quickly by laboratory standards. For some patients, the body ran ahead of the numbers. When the curves rose on our screen, they carried more than science. They carried the echo of the waiting we had all been doing.

Later analyses teased out the context. Podor had no sentinel site in the national syndromic surveillance network before the outbreak (*7*). In at least 1 fatal case, viral hemorrhagic fever was not among the diagnostic considerations at first, suggesting late recognition and confirmation. After the outbreak, sentinel coverage expanded to Podor (*6*). The science is clear, but the human lesson is clearer: surveillance and training shorten the wait, and sometimes that difference is a life.

We returned to our benches the next day. The machines hummed, the city stirred, and somewhere a family decided how to remember those last hours, the hours before the answer. An answer did come, of course, but not soon enough for everyone.

## The Human Weight of Waiting

Ebola and Crimean-Congo hemorrhagic fever are different diseases, and the outbreak scenarios for each comprised different ecologies and fears. Still, between sample and answer lies the same fragile interval. 

For families, waiting corrodes trust. Science can feel like the barrier between love and ritual, between mourning and the ground. For clinicians, waiting corrodes confidence. Hands hover over a patient because confirmation carries moral and occupational weight. For scientists, waiting corrodes certainty. Each required cycle takes place inside someone else’s life.

On paper, the outbreak story celebrates speed, rapid sequencing, new assays, quick analyses. In practice, outbreak work is dominated by waiting together: with families at gates, with clinicians in wards, with technicians facing racks that do not end. Even in North Kivu, where field labs reported <48-hour targets for suspect samples, the night-to-morning gap and the realities of transport and insecurity meant that many woke up facing the same uncertainty that followed them to bed. That gap is real, measurable, and human.

Waiting is not absence; it is a charged presence. Medical anthropologists call this liminality (*8*): suspended between states, neither sick nor well, neither bereaved nor released. In that space, imagination does its worst and best, hope and dread balancing on the same fingertip. The ethical demand is simple to describe but difficult to meet: shorten the wait and empathize with those who are waiting. Achieving this initiative would mean investments in specimen transport and lab networks, decentralization when possible, transparent communication about where a sample is located and when a result is likely, and above all, acknowledging that every “pending” is a person.

## Not Always in Time

The machine always finishes eventually. In Beni, the result authorized a burial, but it could not erase the anger shaped by hours outside a locked door. In Podor, confirmation guided the response and strengthened the system, but 2 families had already crossed into mourning.

This is the hardest truth I learned at the bench and at the door: the result always comes, but it does not always arrive in time. The philosophy of waiting in outbreak science is simply this: precision owes a debt to compassion. Between sample and answer, we either pay that debt together or we collect it in grief.
